# Supplementary material for: Surface chemical heterogeneous distribution in over-lithiated Li1+xCoO2 electrodes
Source: Nat Commun. 2022 Oct 29;13:6464. doi: 10.1038/s41467-022-34161-4 (PMC9617898; doi:10.1038/s41467-022-34161-4)
Supplement: Supplementary file 1 — Supplementary Information [file 41467_2022_34161_MOESM1_ESM.pdf]

*Supporting information*

# Surface chemical heterogeneous distribution in over-lithiated $Li_{1+x}CoO_2$ electrodes

Gang Sun [1, 2#], Fu-Da Yu [3#], Mi Lu[4], Qingjun Zhu [1], Yunshan Jiang [5], Yongzhi Mao [5], John A. McLeod [6], Jason Maley [7], Jian Wang [8*], Jigang Zhou [8*], Zhenbo Wang [1, 5*]

[1] College of Materials Science and Engineering, Shenzhen University, Shenzhen 518071, China
[2] College of Physics and Optoelectronic Engineering, Shenzhen University, Shenzhen 518060, China
[3] College of Material Science and Engineering, Huaqiao University, Xiamen 361021, China
[4] Key Laboratory of Functional Materials and Applications of Fujian Province, School of Materials Science and Engineering, Xiamen University of Technology, Xiamen 361024, China
[5] School of Chemistry and Chemical Engineering, Harbin Institute of Technology, Harbin 150001, China, E-mail: wangzhb@hit.edu.cn;
[6] Department of Electrical & Computer Engineering, Western University, London, ON N6A 5B9, Canada
[7] Department of Chemistry and Saskatchewan Structural Sciences Centre, University of Saskatchewan, 110 Science Place, Saskatoon, Saskatchewan S7N 5C9, Canada
[8] Canadian Light Source Inc., University of Saskatchewan, Saskatoon, SK S7N 2V3, Canada, E-mails: jian.wang@lightsource.ca; jigang.zhou@lightsource.ca;

These authors contributed equally: Gang Sun, Fu-Da Yu.

These authors jointly supervised this work: Jian Wang, Jigang Zhou, Zhenbo Wang

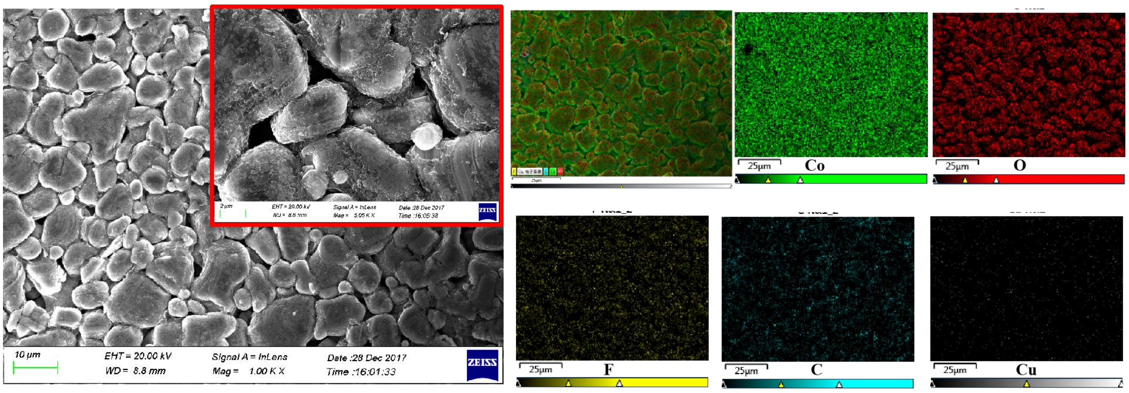


Figure S1 SEM images and the corresponding EDS mapping at Co, O, F, C, and Cu of an overdischarged $LiCoO_2$ electrode (D-0.0V).

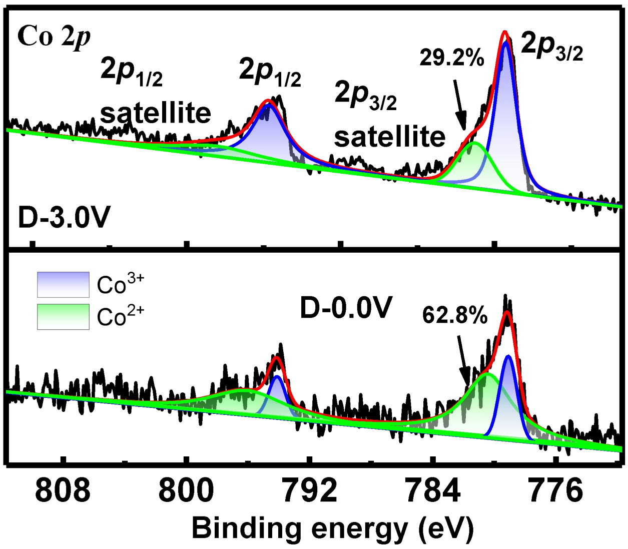


Figure S2 Core-level XPS spectra of Co 2*p* of D-3.0V and D-0.0V electrodes.

## The details of O K-edge XANES simulation

Reference spectra from CoO, $Co_3O_4$, $CoO_2$, $Li_2O$, and $LiCoO_2$ were calculated based on reported crystal structures[1-5]. These structures were obtained from the Crystallography Online Database (www.crystallography.net). DFT is probably most often performed using the Perdew, Burke, and Ernzerhof (PBE) generalized gradients approximation (GGA) for the exchange-correlation potential[6]. This method is known to underestimate band gaps, and often

performs poorly for transition metal-oxide compounds. Since most of these compounds involve cobalt bonded to oxygen, it is important to test the influence of magnetic order and correlated 3*d* electrons. Adding a “Hubbard U” potential to the metal 3*d* orbitals is a popular technique (DFT+U or GGA+U), but here we used the more modern modified Becke-Johnson exchange-correlation potential (mBJ) that has previously demonstrated good agreement with experiment for several metal oxides, and does not require an arbitrary choice of the value for the U potential[7].

These core-hole calculations were performed only with the PBE exchange-correlation function (PBE+CH). Very conveniently, the literature suggests that the $Co^{3+}$ in $LiCoO_2$ is in a S = 0 spin state, so magnetic order does not need to be considered[8]. Furthermore, the literature suggests that including effects of correlated 3*d* electrons is not necessary (i.e. DFT+U or mBJ gives same results as just PBE)[9]. Finally, the literature suggests that core-hole screening is underestimated in $LiCoO_2$, so while including the core-hole (i.e. PBE+CH or even mBJ+CH) improves the overall energy alignment (relative to the valence band), spectral features are somewhat more accurate without the core-hole. The calculations confirm the suggestions in the literature: $LiCoO_2$ is not magnetic: if the calculation is initialized with antiferromagnetic ordering with $Co^{3+}$ in the high spin state, the magnetic moments are reduced to zero as the calculation converges to the solution. The calculations also show that the core-hole effect is minimal in regards to the shape of the absorption spectrum for all Co-based materials.

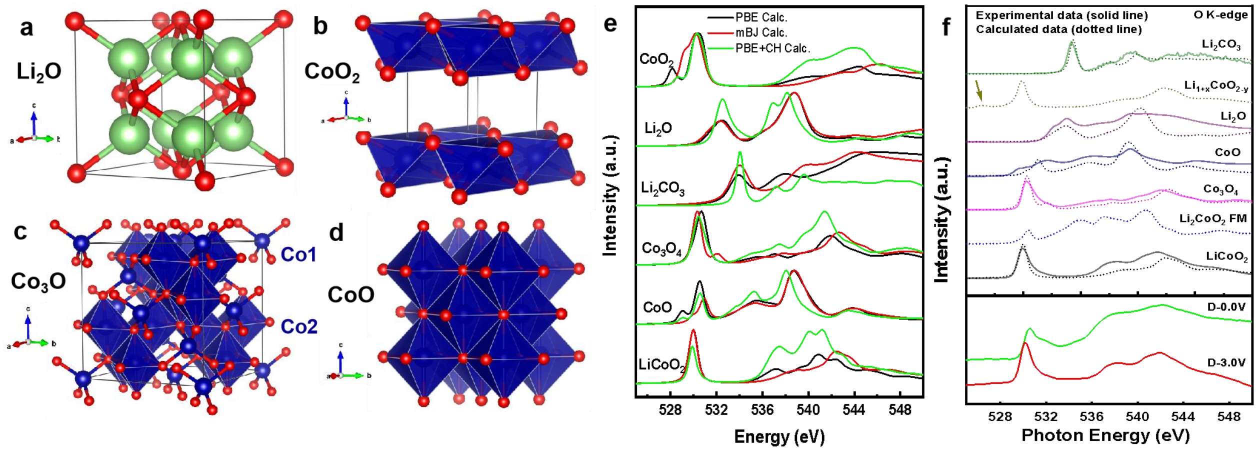


Figure S3 The geometrical configurations of $Li_2O$ (a), $CoO_2$ (b)$Co_3O_4$, CoO (d); (e) Calculated O K-edge spectra for $Li_2O$, $CoO_2$, CoO, $Co_3O_4$ and $LiCoO_2$ using PBE, mBJ, and PBE+CH methods. Top: Calculated O K-edge XANES spectra for $CoO_2$, $Co_3O_4$, $Li_2O$+CoO, $Li_2O$, CoO, $Li_{1+x}CoO_{2-y}$, $Li_2CoO_2$, and $LiCoO_2$ using the mBJ method. bottom: O K-edge XANES of D-3.0V and D-0.0V samples;

The calculated spectra are shown in Figure S3. The energy alignment for each spectrum may not be accurate and should be shifted to align with experimental measurements. The spectral broadening is also somewhat arbitrarily chosen and can be adjusted to better match measurements. Qualitatively it looks like the non-core-hole mBJ calculations are a slightly better match to the measured data. Based on this, we used only mBJ to calculate XANES for the more complicated over-stoichiometric $Li_xCoO_2$ system.

The experimental O K-edge XANES of CoO, $Li_2O$, $Co_3O_4$, $LiCoO_2$ and $Li_2CO_3$ were also added in Figure S3f to compared that of experimental discharge/over-discharged electrode. In addition, since $Li_2CoO_2$ and $Li_{1+x}CoO_{2-y}$ has no real object as a reference, only calculated O K-edge spectrum can be provided as a reference. Here, calculated the O spectrum of CoO, $Li_2O$, $Co_3O_4$, $LiCoO_2$ and $Li_2CO_3$ materials are provided in Figure S3e and S3f for comparison with the experimental spectrum. Since the reference material may have impurities, defects and other shortcomings, there are certain differences between the experimental and calculated O spectra, but it needs to be emphasized that the calculated spectra well replicated all the main features of the experimental spectra from the reference compounds (Check the photon energy between this and the manuscript).

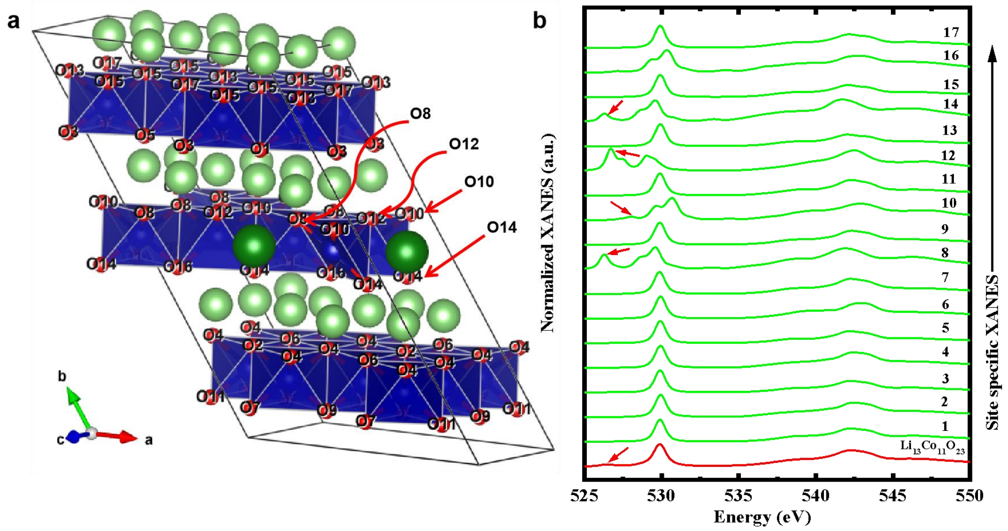

Figure S4 (a) The geometrical configurations of $Li_{13}Co_{11}O_{23}$; (b) Calculated O K-edge spectra for over-stoichiometric $Li_{13}Co_{11}O_{23}$ using mBJ. The $Li_{13}O_{11}O_{23}$ has 17 unique oxygen sites, the individual XANES for each are shown in green (the sum of all XANES is shown in red).

The calculated spectra for $Li_{13}Co_{11}O_{23}$ are shown in Figure S4, with the XANES spectrum from each unique O site shown separately. A measurement should sample all sites equally, so the average XANES of all O sites is what is actually measured. However, if the actual stoichiometry is different from the calculated one (as is probably the case), knowing the XANES of each individual site can help simulate a more accurate calculated spectrum by changing the relative weight in each site. It is clear that the oxygen K-edge XANES spectrum from most sites is almost identical to that of pristine $LiCoO_2$. The most distorted spectra are the ones adjacent to the substituted Li and the oxygen vacancy (namely sites 8, 12, and 14). Unfortunately, these spectra do not agree well with the measured data, as the measurements show a reduction in the pre-edge peak, not an enhancement in features at even lower energies.

Furthermore, the oxygen vacancies will lead to a small peak which appears before 529 eV in the O K-egde XAS, as shown in Figure S4 (As the arrow points.). It is weakly related to the valence state of $Co^{2+/3+}$, as it does not appear in the oxygen spectrum of either calculated or actual CoO and $Co_3O_4$ (containing $Co^{2+}$ ions). The calculated $Li_{1+x}CoO_{2-y}$ spectrum is different from the experimental one, therefore we excluded the oxygen vacancy model.

Furthermore, XANES is dominated by short-range structure because the transition involves the overlap integral of the conduction band states with the core electron's wavefunction (which is obviously highly localized to the absorbing atom). In the present

situation, our $Li_{13}Co_{11}O_{23}$ structure has some crystal symmetry (space group *Bm*) and results in 17 unique oxygen sites. We can break our simulation down into the XANES contribution from each of these sites. Most of these sites (12 out of the 17) provide XANES that are basically identical to that of $LiCoO_2$. The three unique oxygen sites that bond directly to the substituted $Li_{3a}$ all have the low-energy feature at 529 eV. The remaining two are bonded to Co but next to $V_O$, and these have additional fine structure in the main feature at 530 eV, which gets washed out when the contribution from all sites is averaged.

Consequently, our approach is less dependent on stoichiometry (and crystal structure) than it might seem. A full DFT study of a system with vacancies or impurities may require investigating a range of stoichiometries, as well as a range of geometries for each stoichiometry. However if we are only focused on calculating XANES for structures with vacancies or impurities it is necessary to obtain only a structure with a reasonable concentration of these vacancies or impurities, and with a geometry that does not provide extreme clustering of these vacancies or impurities (i.e., in the present case, a $Li_xCoO_y$ structure with an entire $CoO_2$ layer replaced with Li substituents and O vacancies is obviously extreme).

When the result shows that most of the site-specific spectra match those of the stoichiometric system (as is the case here), we can be reasonably confident that there is negligible interaction in the XANES spectra between the introduced defects. Consequently, we can construct a "background" XANES from all sites that match the stoichiometric system, and a "$Li_{3a}$+$V_O$" XANES from the specific sites that show distortion, and then linearly rescale the intensities these simulated XANES to investigate other stiochiometries (again, as long as the concentration of vacancies or impurities does not become so large that strong interactions between adjacent defects will have a significant effect). We do not quantitatively investigate this in the present paper because it is clear that in order to reproduce the measured spectra the "$Li_{3a}$+$V_O$" contribution to the XANES would need to be scaled to zero, as there is no feature in the measured data near 529 eV, and no new features from the "$Li_{3a}$+$V_O$" contribution new 538 eV (where the measured data most strongly differs from the spectra from stoichiometric $LiCoO_2$).

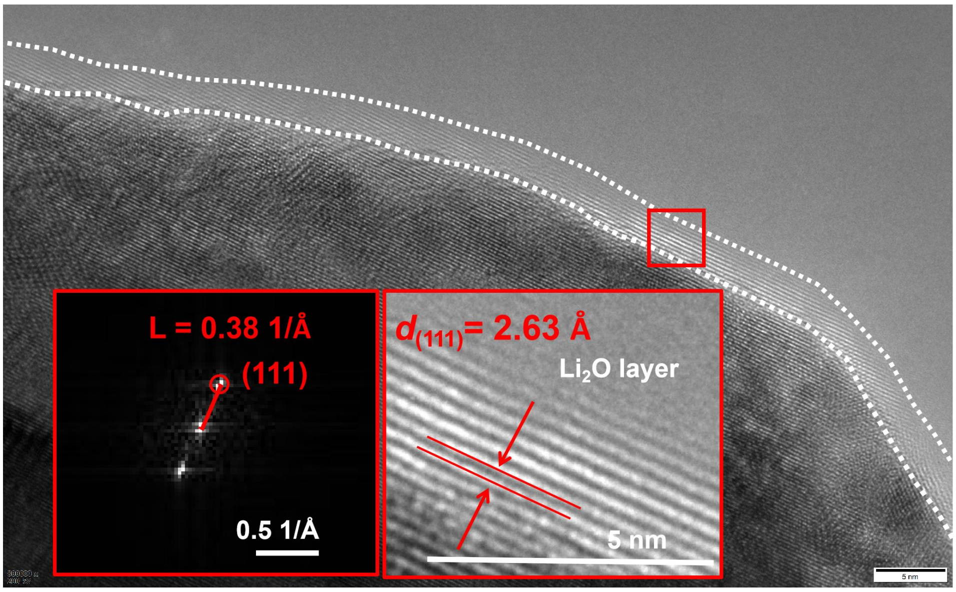


Figure S5 HRTEM and the corresponding FT/IFT images of D-0.0V electrode ($Li_2O$-like phase).

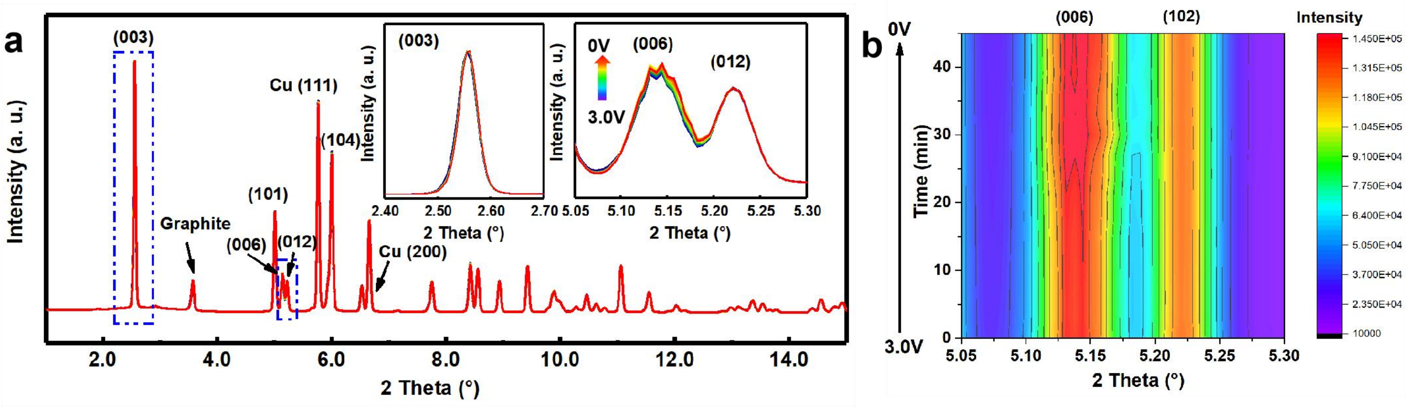


Figure S6 In-situ synchrotron XRD of the overdischarged $LiCoO_2$/graphite pouch cell (Discharge from 3.0V to 0.0V at 20 mA).

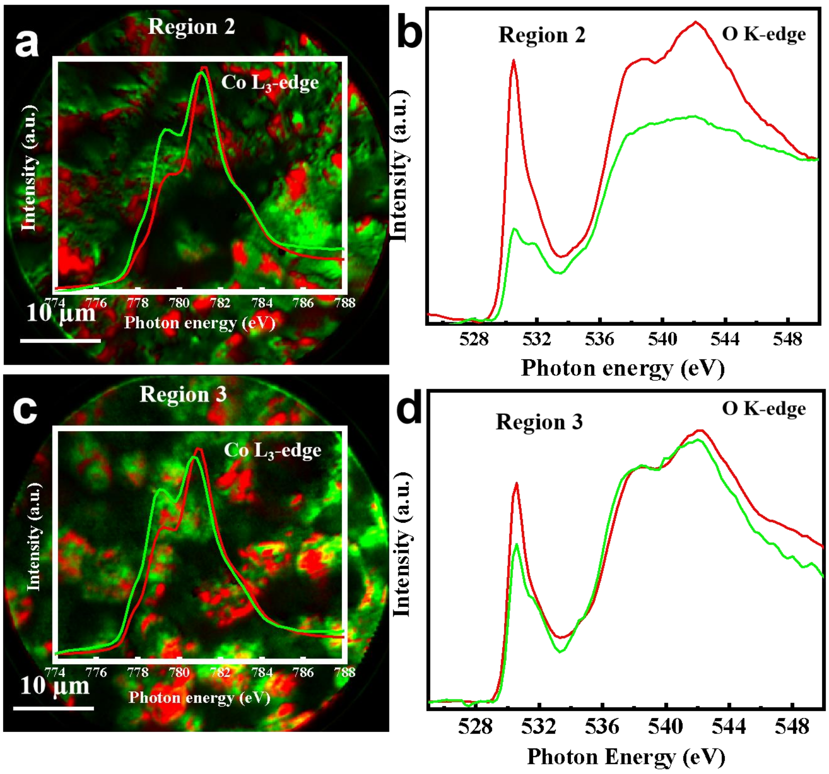


Figure S7 Co chemical mapping based on fitting Co $L_3$-edge image stacks (illustrations are Co $L_3$-edge XANES of red and green regions) of the D-0.0V electrode at two regions and the corresponding O K-edge XANES of red and green regions. Region 1 (a, b); Region 2(c, d).

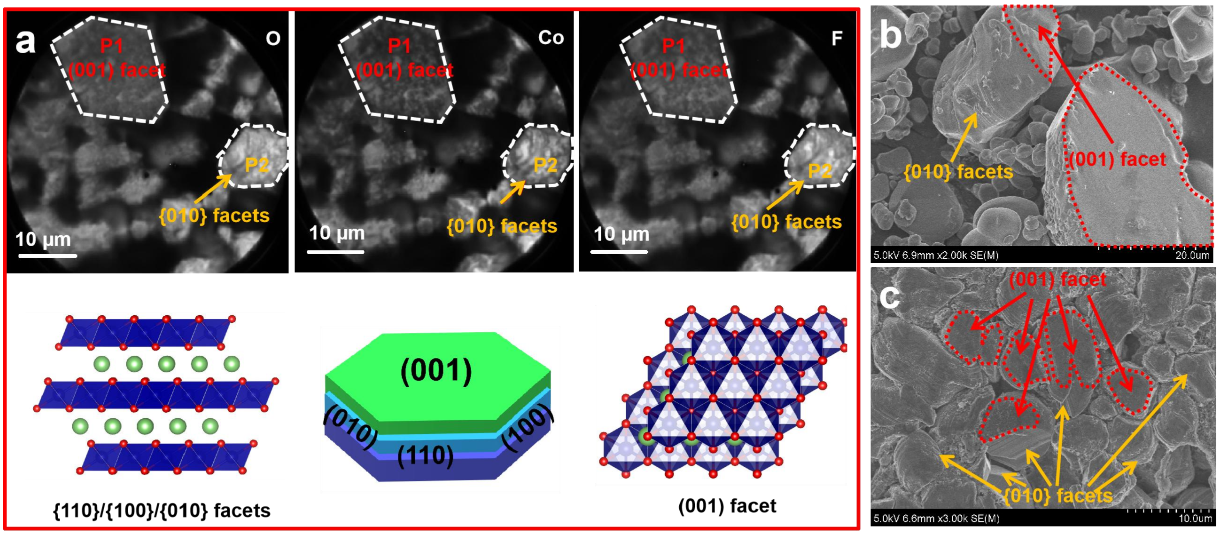


Figure S8 (a) O, Co and F mapping of the D-0.0V electrode by X-PEEM, together with the schematic illustrations of the crystal shape with different crystal planes of a $LiCoO_2$ crystal. (b, c) The SEM images of pristine $LiCoO_2$ particles and D-0.0V electrode, respectivily.

Additional XRD, SEM and HRTEM data were further probed and analyzed in order to better understand the exposed crystal planes of the pristine $LiCoO_2$ particles and the surface structure information of the overdischarge electrode. The sXRD data in Figure S6 show that the peak intensity of (003) is the strongest, indicating that (003) is the dominant crystal plane for particle growth (the grain has the priority to growth along the c-axis, [001] direction). In addition, the lateral planes of the (003) facet would be wrapped by the {010}/{100}/{110} facets, so the (003) facet and the {010}/{100}/{110} facets would be preferentially exposed on the single crystal grains of $LiCoO_2$. The SEM images of the pristine $LiCoO_2$ particles (Figure S8b) and D-0.0V electrode (Figure S8c) also reveal these large single crystal primary particles with different exposed facets. The SEM images delineate step edges, as expected for layered $LiCoO_2$ grains (Figure S8b and S8c), and a similar phenomenon is observed on P2 in Figure 3a and 3b. The particles with a smooth surface and extended sidewalls suggest that the particle has an exposed (001) facet (as marked regions in red dotted line in SEM images). This conclusion has also been confirmed in the previous literature[10, 11]. At the same time, multiple particles were explored on the D-0.0V electrode in Figure S8c to achieve better statistical data. Besides, the obvious step edges and lamellar shapes, marked in yellow arrows in Figure R10, capped by a (001) facet, parallel to the c-axis, indicating that the exposed crystalline facets belong to the {010}/{100}/{110}facets.

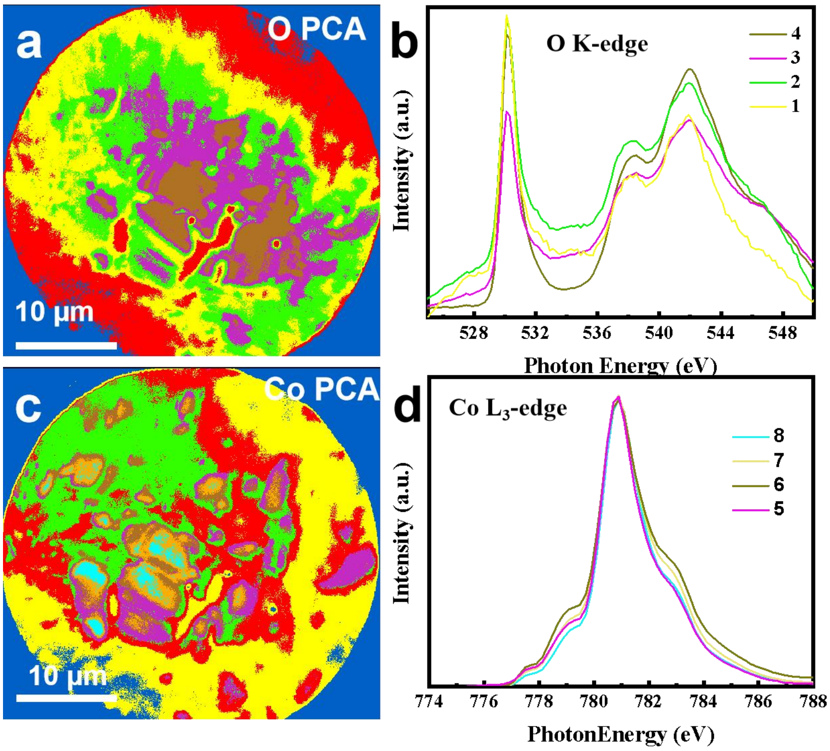


Figure S9 (a) O edge PCA and (c) Co edge PCA analysis of a discharged D-3.0V electrode; corresponding O K-edge (b) and Co $L_3$-edge (d) XANES spectra extracted at virous color regions from (a and c).

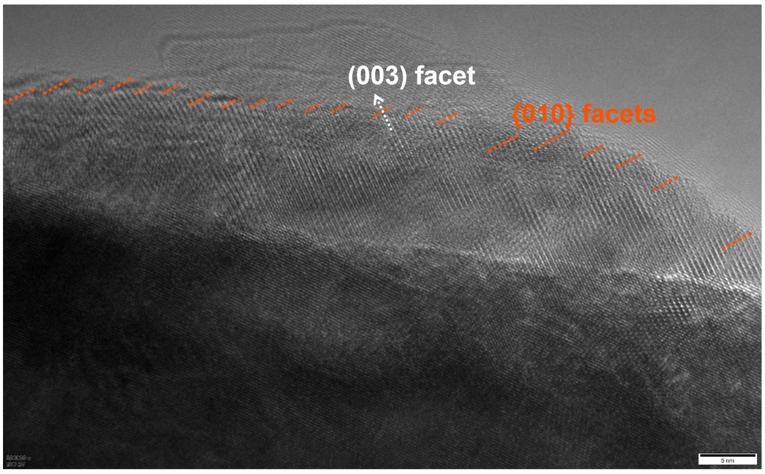


Figure S10 HRTEM image of D-0.0V electrode.

**Calculation details for the electronic structures and ionic diffusion of $LiCoO_2$ and $Li_2CoO_2$ modes**

Theoretical calculations of the lattice relaxations, electronic structures, and ionic diffusion were performed by using the CASTEP module of Materials Studio software package within the DFT framework[12]. Spin-polarized version of the PBE functional under generalized gradient approximation (GGA-PBE) was adopted to treat the exchange correlation energy with the projector augmented wave (PAW) potentials. Due to the strongly correlated 3d-electrons, the rotationally invariant approach (DFT+U) was introduced[13], and the Hubbard U of Co was set to 3.32 eV, according to previous literature[14]. It should be pointed out that the applied U will significantly affect the total energies of the systems, especially for the structures with different lithium contents. However, by using the same parameters, qualitative and meaningful results can still be achieved. The plane-wave energy cutoff and the self-consistent field tolerance were set to 500 eV and $1.0\times10^{-6}$ eV·atom$^{-1}$, respectively, with a Gamma-centered 5×5×3 Monkhorst-Pack mesh being applied to deal with the numerical sampling over the Brillouin zone. The atomic coordinates and lattice parameters were fully relaxed during the geometry optimization process, and iterations were repeated until the energy and force acting on each atom is less than $1.0\times10^{-6}$ eV·atom$^{-1}$ and 0.01 eV·Å$^{-1}$, respectively. Due to considerations of precision and calculation time, supercells of $Li_{27}Co_{27}O_{54}$ (composed of 27 units of $LiCoO_2$), and over-lithiated $Li_{54}Co_{27}O_{54}$ (composed of 27 units of $Li_2CoO_2$) were used for the calculation of the $Li^+$ diffusion. The minimum energy pathways of the $Li^+$ diffusion from one lattice site to the adjacent ones were investigated by the climbing image nudged elastic band (CI-NEB) method. The internal atomic positions of the initial and final structures were both optimized before the CI-NEB calculations.

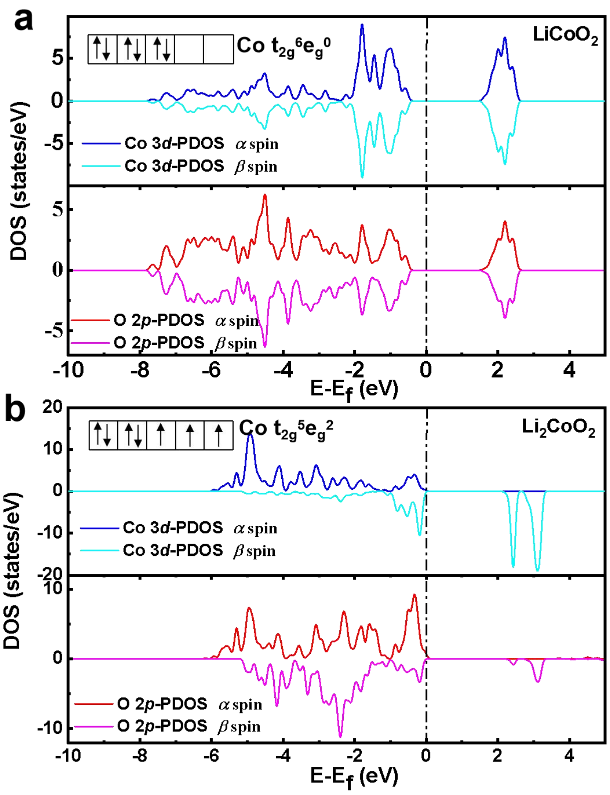


Figure S11 Computed Co-3*d* and O-2*p* PDOS of (a) $LiCoO_2$ and (b) $Li_2CoO_2$, $E_f$ represents the Fermi level.

As displayed in Figure S11, for $LiCoO_2$, the initial spin configuration of the Co-3*d* states is set to $t_{2g}^6e_g^0$, namely, the $t_{2g}$ states ($t_{2g}^6$) are fully occupied, and the $e_g$ states are unfilled, which has a theoretical magnetic moment of 0 $\mu b$. The PDOS of the Co and O atom exhibits almost identical $\alpha$ and $\beta$ spin channels, indicating zero net magnetic moments. On the other hand, the initial spin configuration of the Co-3*d* states in $Li_2CoO_2$ is set to $t_{2g}^5e_g^2$. The PDOS of $Li_2CoO_2$ shows that the conduction band of Co-3*d* states is fully provided by $\beta$ spin channels, leading to a calculated spin magnetic moment of 2.68 $\mu_b$, which is consistent with the $t_{2g}^5e_g^2$ spin configuration.

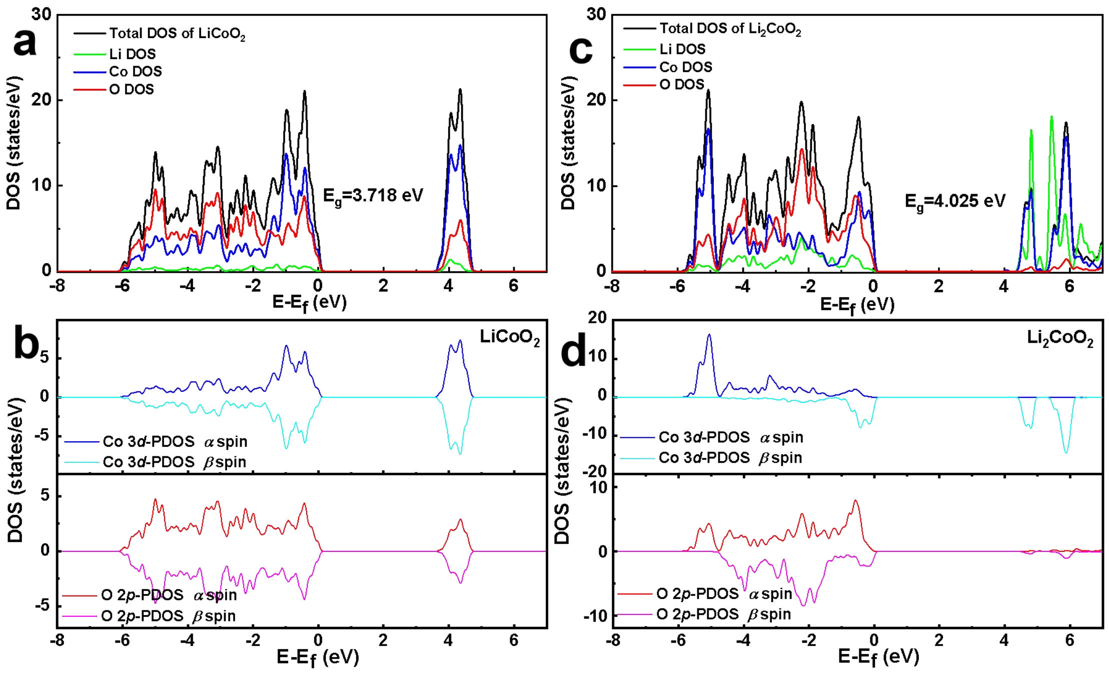


Figure S12 The total density of states (DOS) and the partial density of states (pDOS) of $LiCoO_2$ (a) and $Li_2CoO_2$ (c); Computed Co-3*d* and O-2*p* PDOS of $LiCoO_2$ (b) and $Li_2CoO_2$ (d).

To obtain accurate electronic structures, we added theoretical calculations using the Heyd-Scuseria-Ernzerhof (HSE06) screened hybrid functional, in which an amount of exact Hartree-Fock exchange mixing parameter of 0.25 is used, and the results are shown in Figure S12. We show that the HSE06 functional yields larger band gaps for $LiCoO_2$ and $Li_2CoO_2$ as compared to GGA+*U* because it can eliminate self-interaction error, resulting in an over-delocalization of the electron. The hybrid functional calculation result of the band gap for $LiCoO_2$ is in good agreement with the literature[15]. However, as expected, the theory level impacts the band gap amplitude but not the global shape of the electronic band structures.

# References


1. Sasaki, S., Fujino, K. & Takéuchi, Y. X-ray determination of electron-density distributions in oxides, MgO, MnO, CoO, and NiO, and atomic scattering factors of their constituent atoms. *Proceedings of the Japan Academy, Series B*. **55**, 43-48 (1979).
2. Roth, W. L. The magnetic structure of Co3O4. *J. Phys. Chem. Solids*. **25**, 1-10 (1964).
3. Tarascon, J. M. et al. In situ structural and electrochemical study of Ni1− xCoxO2 metastable oxides prepared by soft chemistry. *J. Solid State Chem.* **147**, 410-420 (1999).
4. Wyckoff, R. Crystal Structures, Interscience. *New York*. **1**, 254 (1963).
5. Lin, Q., Li, Q. A., Gray, K. E. & Mitchell, J. F. Vapor growth and chemical delithiation of stoichiometric LiCoO2 crystals. *Cryst. Growth Des.* **12**, 1232-1238 (2012).
6. Perdew, J. P., Burke, K. & Ernzerhof, M. Generalized gradient approximation made simple. *Phys. Rev. Lett.* **77**, 3865 (1996).
7. Tran, F. & Blaha, P. Accurate band gaps of semiconductors and insulators with a semilocal exchange-correlation potential. *Phys. Rev. Lett.* **102**, 226401 (2009).
8. Vallverdu, G., Minvielle, M., Andreu, N., Gonbeau, D. & Baraille, I. First principle study of the surface reactivity of layered lithium oxides LiMO2 (M= Ni, Mn, Co). *Surf. Sci.* **649**, 46-55 (2016).
9. Kalantarian, M. M., Asgari, S. & Mustarelli, P. A theoretical approach to evaluate the rate capability of Li-ion battery cathode materials. *J. Mater. Chem. A*. **2**, 107-115 (2014).
10. Balke, N. et al. Nanoscale mapping of ion diffusion in a lithium-ion battery cathode. *Nat. Nanotechnol.* **5**, 749-754 (2010).
11. Wu, N. et al. Flakelike $LiCoO_2$ with Exposed {010} Facets As a Stable Cathode Material for Highly Reversible Lithium Storage. *ACS Appl. Mater. Inter.* **8**, 2723-2731 (2016).
12. Clark, S. J. et al. First principles methods using CASTEP. *ZEITSCHRIFT FUR KRISTALLOGRAPHIE*. **220**, 567-570 (2005).
13. Richards, W. D., Dacek, S. T., Kitchaev, D. A. & Ceder, G. Fluorination of Lithium-Excess Transition Metal Oxide Cathode Materials. *Adv. Energy Mater.* **8**, 1701533 (2018).
14. Ben Yahia, M., Vergnet, J., Saubanère, M. & Doublet, M. Unified picture of anionic redox in Li/Na-ion batteries. *Nat. Mater.*, (2019).
15. Seo D H, Urban A, Ceder G. Calibrating transition metal energy levels and oxygen bands in first principles calculations: accurate prediction of redox potentials and charge transfer in lithium transition metal oxides. *Phys. Rev. B*, **92**, 115118 (2015).
